# Evaluation of *In Vitro* Cytoxicity and Genotoxicity of Size-Fractionated Air Particles Sampled during Road Tunnel Construction

**DOI:** 10.1155/2013/345724

**Published:** 2013-08-28

**Authors:** Luca Dominici, Elena Guerrera, Milena Villarini, Cristina Fatigoni, Massimo Moretti, Paolo Blasi, Silvano Monarca

**Affiliations:** ^1^Dipartimento di Specialità Medico-Chirurgiche e Sanità Pubblica (Sezione di Sanità Pubblica), Università degli Studi di Perugia, Via del Giochetto, 06122 Perugia, Italy; ^2^Istituto Nazionale per l'Assicurazione contro gli Infortuni sul Lavoro (INAIL), Direzione Regionale Umbria, Consulenza Tecnica Accertamento Rischi e Prevenzione (CONTARP), Via G. Battista Pontani, 06128 Perugia, Italy; ^3^Dipartimento di Chimica e Tecnologia del Farmaco (Sezione di Tecnologie Farmaceutiche), Università degli Studi di Perugia, Via del Liceo, 06123 Perugia, Italy

## Abstract

In tunnel construction, workers exposed to dust from blasting, gases, diesel exhausts, and oil mist have shown higher risk for pulmonary diseases. A clear mechanism to explain how these pollutants determine diseases is lacking, and alveolar epithelium's capacity to ingest inhaled fine particles is not well characterized. The objective of this study was to assess the genotoxic effect exerted by fine particles collected in seven tunnels using the cytokinesis-block micronuclei test in an *in vitro* model on type II lung epithelium A549 cells. For each tunnel, five fractions with different aerodynamic diameters of particulate matter were collected with a multistage cascade sampler. The human epithelial cell line A549 was exposed to 0.2 m^3^/mL equivalent of particulate for 24 h before testing. The cytotoxic effects of particulate matter on A549 cells were also evaluated in two different viability tests. In order to evaluate the cells' ability to take up fine particles, imaging with transmission electron microscopy of cells after exposure to particulate matter was performed. Particle endocytosis after 24 h exposure was observed as intracellular aggregates of membrane-bound particles. This morphologic evidence did not correspond to an increase in genotoxicity detected by the micronucleus test.

## 1. Introduction

The relationship between exposure to fine particulate air pollution and acute and chronic effects (e.g., lung cancer), especially in combination with other known risk factors, such as occupational exposures, is well documented [1–3].

Workers during tunnel construction are exposed to airborne complex mixtures of many toxic agents (e.g., nitrogen dioxide, silica, cement, oil mist, and diesel exhaust); some of them are known to be genotoxic and cause respiratory disease and cancer. Research on the health impact of this occupational exposure has shown in tunnel workers decreased lung function, increased incidence of respiratory inflammation, and bronchial hyperresponsiveness [4–9]. Several studies suggest that there is an elevated risk of lung cancer for workers exposed to elevated levels of dust from crushed stone facilities, and exposition to silica and diesel particulate matter constitutes the main sources of risk [10–14].

The typical lung reaction induced by chronic inhalation of crystalline silica is silicosis, a generally progressive fibrotic lung disease (pneumoconiosis) [15], and however, with the association of crystalline silica (mainly quartz) exposure and silicosis, as well as lung cancer, chronic obstructive pulmonary disease and pulmonary tuberculosis were reported [16]. In 1997, the International Agency for Research on Cancer (IARC) classified some crystalline silica polymorphs (i.e., quartz and cristobalite) in Group 1 (carcinogenic to humans), whereas amorphous silica (i.e., silicon dioxide without crystalline structure) was classified in Group 3 (not classifiable as to its carcinogenicity for humans) [17].

Tunnel workers are also exposed to diesel exhaust that showed in different working environments a strong and consistent relationship with an increased risk of dying from lung cancer [18]. In 2012, IARC has classified diesel engine exhaust in Group 1 [19]. Diesel exhaust particles, diesel exhaust condensates, and organic solvent extracts of diesel engine exhaust particles induced, *in vitro* and *in vivo*, various forms of DNA damage, including bulky adducts, oxidative damage, strand breaks, unscheduled DNA synthesis, mutations, sister chromatid exchanges (SCE), morphological cell transformation in mammalian cells, and mutations in bacteria [19]. Positive genotoxicity biomarkers of exposure and effect were also observed in humans exposed to diesel engine exhaust.

Very few studies have been conducted to evaluate genotoxic hazard for workers employed in tunnel construction. A previously published molecular epidemiology study [20] was designed to evaluate whether occupational exposure during road tunnel construction might result in genotoxic effects. Different genotoxicity tests were carried out in leucocytes of exposed and control subjects. There were no significant differences in the level of primary and oxidative DNA damage (comet assay) and frequency of SCE between the tunnel workers and controls, whereas the frequency of micronuclei (MN) showed a significant increase in exposed subjects compared to controls.

Experimental studies have shown that smaller particles induce stronger biological effects than larger particles of similar composition, due to their larger surface area to mass-ratio [21], and several researches have shown that the size of the airborne particles and their surface area determine the potential to elicit inflammatory injury, oxidative damage, and other biological effects. These effects are stronger for fine and ultrafine particles because they can penetrate deeper into the airways of the respiratory tract and can reach the alveoli [3].

Atmospheric submicron particles, such as ultrafine particles (UFP, aerodynamic diameter ≤ 100 nm) and particulate matter ≤ 1.0 *μ*m (PM 1.0), are an emerging important health threat to humans. In particular, UFP show high toxicity, since they carry considerable amounts of air toxicants [22] and have recently shown to promote vascular oxidative stress, vascular calcification, and inflammatory responses [23]. UFP have been shown to cause genotoxic effects, and oxidative stress and proinflammatory events seemed to be a generic characteristic in a number of *in vitro* and *in vivo* models [24, 25].

There have been few studies on genotoxicity of submicron particles in urban atmosphere: Monarca et al. have shown that PM with aerodynamic diameter ≤ 0.5 *μ*m (PM 0.5) contains most of the airborne polycyclic aromatic hydrocarbons (PAH) and shows genotoxicity [26]. These data were confirmed by Massolo et al. in different urban airborne particulates [27]. More recently, Topinka et al. have found that UFP of various ambient-air samples is neither a major carrier of PAH nor a major inducer of their genotoxicity, whereas the submicron ambient-air particle fraction, as a whole, is a carrier of 80–90% of total PAH and of 70–80% of total air particulate genotoxicity [28].

Although these studies suggest the particular hazards due to the human exposure to urban air submicron particles, no similar studies have been carried out in underground occupational environments. The development of new size-selective samplers and pump technology has enhanced the ability to evaluate PM exposure levels based on new occupational criteria.

The focus of this paper was the hazard identification of size-fractionated particles sampled during tunnel construction by a size-selective sampler. Particulate fractions were studied by means of *in vitro* genotoxicity and cytoxicity tests. Analyses by SEM and TEM microscopes of the particle morphology and migration in human pulmonary cells were also carried out.

## 2. Materials and Methods

### 2.1. Chemicals

All reagents used were of analytical grade. Acetic acid, Giemsa stain solution, methanol, ethanol, propylene oxide, potassium chloride (KCl), disodium phosphate (Na_2_HPO_4_), and monobasic potassium phosphate (KH_2_PO_4_) were purchased from Carlo Erba Reagenti, Milan, Italy. Glutaraldehyde, sodium cacodylate, osmium tetroxide, ethyl methanesulfonate (EMS), fluorescein-diacetate (FDA), and propidium iodide (PI) were obtained from Sigma-Aldrich Srl, Milan, Italy. F-12 medium, fetal bovine serum (FBS), cytochalasin B, trypsin-EDTA, antibiotics (penicillin and streptomycin), and Dulbecco's phosphate-buffered saline pH 7.4 (PBS) were purchased from Invitrogen Srl, Milan, Italy. Conventional microscope slides and coverslips were supplied by Knittel-Glaser, Braunschweig, Germany. Eukitt was from O. Kindler GmbH, Freiburg, Germany. Epoxy resin Epon-Araldite was from Fluka, Buchs, Switzerland. Distilled water was used throughout the experiments.

### 2.2. Size-Fractionated Particle Sampling during Tunnel Construction

Airborne particulates were sampled in seven tunnels during their excavation in Central Italy using a multistage cascade sampler (Sioutas Cascade Impactor, SKC, Eighty Four, PA, USA). The samples consist of four impaction stages (A to D) and an after-filter (AF) allowing separation and collection of airborne particles in five size ranges. A pump maintained a constant flow rate of 9 L/min during sampling. The particles were collected on PTFE filters (ø = 25 mm), for the stages A–D and 37 mm for the stage AF. Particles were collected in 5 different fractions with the following aerodynamic diameters: A ≥ 2.5 *μ*m, B = 1.0 to 2.5 *μ*m, C = 0.5 to 1.0 *μ*m, D = 0.25 to 0.50 *μ*m, and AF ≤ 0.25 *μ*m. The samplers were used for area sampling attached to tripods in locations which have been chosen to represent typical job sites and representative particle sources. Sampling sessions were conducted by officers of INAIL/CONTARP (Italian Workers' Compensation Authority—Technical Advisory Department for Risk Assessment and Prevention). At the end of sampling, the filters were removed, dried in a darkened desiccator for 48 h before weighing for gravimetric analysis, stored in appropriate sealed containers, labeled and transported to the laboratory of the Department of Medical-Surgical Specialties and Public Health of the University of Perugia, and kept refrigerated at −20°C until extraction.

### 2.3. Separation of Particles from the Membranes

For the aqueous extraction of fractions, the filters were immersed in complete F-12 medium and sonicated for 30 minutes in an ultrasonic bath to obtain suspension of particles for cell exposure. The sealed containers used for transport of filters were washed with the same medium, and the two aliquots were pooled. The total volume of F-12 medium was determined in order to obtain in used culture plates air volumes corresponding to 0.2 m^3^ equivalent/mL. All operations were carried out under sterile hood, and the extracts were aliquoted for subsequent testing and stored at −20°C until use.

### 2.4. Cell Line, Culture Condition, and Cell Treatment

A549 lung carcinoma type II derived cells (ATCC CCL-185) were obtained from Istituto Zooprofilattico Sperimentale della Lombardia e dell'Emilia Romagna “Bruno Ubertini,” Brescia, Italy. The cells were grown as monolayer cultures in F-12 medium supplemented with 10% (v/v) FBS, 100 units/mL penicillin, and 0.1 mg/mL streptomycin at 37°C in a humidified atmosphere containing 5% CO_2_. A549 cells were subcultured by dispersal with 0.05% trypsin in 0.02% Na_4_EDTA for a contact time of 5 min and replated at a 1 : 2 dilution, which maintained cells in the exponential growth phase. All experiments were performed on A549 cells at passages between 101 and 108. Cell stocks were routinely frozen and stored in liquid N_2_.

A549 cells were grown in 6-well tissue culture plates (Orange Scientific, Braine-l'Alleud, Belgium) at an initial concentration of 5 × 10^5^ cells/well (5 mL/well). After 24 h of incubation, the medium was removed from each well and was replaced by fresh complete growth medium. To assay cytotoxic and genotoxic properties of tunnel airborne particulates, a suspension protocol was employed, in order to test simultaneously the aqueous extract of particles and particles themselves. Briefly, in cell cultures, the medium was replaced with complete F-12 medium containing particles, achieved as described in Section 2.3. Cells were exposed to particle suspensions for 24 h. The test concentration was chosen as to be representative of the volume of air breathed during a work shift, that is, assumed to be for construction workers of about 1.4 m^3^/hour of work [29, 30]. 

### 2.5. Cytotoxicity Assays

Immediately, after the *in vitro* treatment, the cells were washed twice with PBS, detached by trypsinization (300 *μ*L of 0.05% trypsin-EDTA, 5 min), and centrifuged for 10 min at 720 ×g.

Cell viability was evaluated either by the trypan blue dye exclusion test and by fluorochrome-mediated viability test. These separate cytotoxicity assays were performed in order to avoid any artificial results due to interference of PM with colorimetric dyes used in cytotoxicity assays [31]. Briefly, for trypan blue dye exclusion test (live cells exclude the dye), 0.2% trypan blue dye was used. The number of viable (uncolored) and dead (colored) cells was counted using an automated cell counter (Countess; Invitrogen, Carlsbad, CA, USA). Viability % was calculated as the ratio of number of viable cells to all cells. For fluorochrome-mediated viability test, cells were stained with FDA and PI [32] and evaluated by visual scoring. Viable cells were fluoresced green, whereas dead cells were indicated by orange-stained nuclei.

### 2.6. Cytokinesis-Block Micronucleus (CBMN) Test

The CBMN test was performed according to the original method [33] with marginal modifications for adaptation to A549 cells. Immediately after the *in vitro* treatment, the medium was removed and replaced by fresh medium containing cytochalasin B (final concentration 6 *μ*g/mL) to inhibit cell division after mitosis. The cells were then incubated further for 30 h. After that the cells were detached by trypsinization, resuspended in hypotonic solution (3 mL of 0.56% KCl) at room temperature, and fixed with 3 mL of Carnoy's reagent (methanol : glacial acetic acid—5 : 1 v/v). The cell suspensions were centrifuged again for 10 min at 720 ×g and resuspended in 6 mL of fixative. Next, the tubes were centrifuged for 10 min, the supernatant discarded, and the cell suspensions dropped on glass slides (two slides per concentration). After drying, the slides were stained with 4% Giemsa in phosphate buffer (0.06 M Na_2_HPO_4_ and 0.06 M KH_2_PO_4_, pH 6.8) for 7 to 8 min, washed with distilled water, air-dried, and finally mounted with Eukitt. Cells were examined for MN at 400x magnification according to previous reports [33]. All MN slide analysis was conducted under blind-scoring conditions. MN were scored in 1,000 binucleated cells (BNC) for each extract of three totally independently experimental set. Positive (2.4 mM, EMS) and negative (F-12 medium) controls were included in each experiment. To investigate the impact of PM on cell proliferation, the nuclear division index (NDI) was determined using the formula [34]:

(1) $$\begin{array}{c} \text{NDI}=\frac{\left[1\times N_{1}\right]+\left[2\times N_{2}\right]+\left[3\times N_{3}\right]+\left[4\times N_{4}\right]}{1,000}, \end{array}$$

where *N*_1_–*N*_4_ represent the numbers of cells with 1 to 4 nuclei, respectively, and 1,000 is the total number of cells scored.

### 2.7. Statistical Analysis

Each result is expressed as the mean ± standard deviation (SD) of three independent experiments. Data obtained were submitted to statistical evaluation using ANOVA univariate test with *post hoc* Bonferroni correction, and the significance was calculated in comparison to the negative (untreated) and positive (EMS) control, respectively. A value of *P* < 0.05 was considered to be statistically significant for evaluated parameters. Increasing of genotoxicity was calculated with fold induction [35] in relation to the frequency of MN in untreated cells. The SPSS (SPSS, Chicago, IL, USA) statistical software program was used for the analyses.

### 2.8. SEM and TEM Investigations

The particles have been investigated by scanning electron microscope (SEM) and transmission electron microscope (TEM) under different conditions. With SEM was possible to observe the shape and size of the granules of material dispersed directly on the filters of the sampler. The TEM electron microscope was used to analyze whether this submicron material could be internalized in the cells. 

#### 2.8.1. SEM

The air particulate, after chrome sputter coating directly made on the polytetrafluoroethylene filter used for sampling, was observed at magnifications up to 100,000x. All the fractions were observed and sample images acquired. SEM observations were performed at the Laboratory of Nanomaterials (L.U.N.A.), University of Perugia, with a Field Emission Scanning Electron Microscope Field Emission (LEO 1525 with Gemini column, Zeiss, Oberkochen, Germany). Energy dispersive X-ray microanalysis for evaluation of heavy metal was performed with EDX (Bruker, MA, USA).

#### 2.8.2. TEM

The fractions chosen for observation were those of minimum (AF: ø < 0.25 *μ*m) and maximum (A: ø > 2.5 *μ*m) aerodynamic diameters. The experimental protocol for sample preparation involved the exposure of A549 cells to air particles resuspended in culture medium (as for genotoxicity testing). Cell pellets were fixed in 2% glutaraldehyde buffered with 0.1 M sodium cacodylate, pH 7.4, for 18 h. The cells were then washed with 0.1 M Sorensen phosphate buffer, pH 7.4, then fixed in 1% osmium tetroxide in 0.1 M phosphate buffer (90 min) and then dehydrated in ascending ethanol series. After the transfer in propylene oxide, the pellets were embedded in epoxy resin. The samples obtained were sectioned by microtome and ultrathin sections (50 nm) observed [36]. The observations were carried out with a Philips TEM 208 (Philips, Eindhoven, Netherlands) at the University Center of Electron Microscopy of Perugia.

## 3. Results

### 3.1. PM Characterization

Table 1 shows PM concentrations for the five fractions sampled by Sioutas multistage cascade impactor in seven tunnels. In all samples, the fraction AF (ø < 0.25 *μ*m) represented about 10–20% of total PM collected. 

Differences in PM composition can be observed for the seven tunnels investigated, with air samples from tunnel 2 having the highest concentration of PM (6.2 mg/m^3^), especially derived from fraction A (ø > 2.5 *μ*m).

In six of seven tunnels, fraction A determined the richest fraction, while in tunnel 7, the fraction AF, in which ultrafine particles under 0.25 *μ*m diameter, was found to have the higher level of concentration (above 2 mg/m^3^). Except for tunnel 2, the level of total PM was always found to be below the concentration of 5 mg/m^3^.

### 3.2. Cytotoxicity Assay

To assess the effects of PM on A549 cell viability, direct cell counting was performed. Percent viability in A549 cells exposed to different air PM fraction was comparable to that observed in controls for both viability tests performed, with a percentage of viability always above 90% (data not shown).

### 3.3. Cytokinesis-Block Micronucleus (CBMN) Test

**Figure 1:**
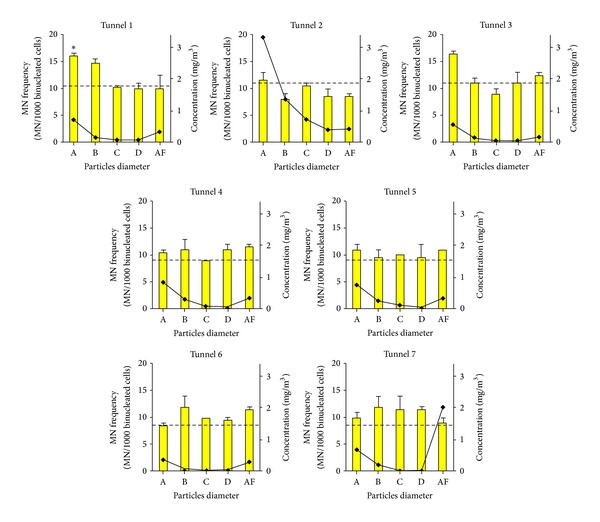
Evaluation of genotoxicity of airborne particles sampled in 7 tunnels under construction in Central Italy on A549 pulmonary cells. Cells were treated for 24 hours with atmospheric extract (mg/m^3^, black points). For each tunnel, genotoxic effects of 5 fractions of different size were evaluated (yellow bars show MN frequency ± SD of each fraction; untreated MN value is shown with dotted line). *P < 0.05 ANOVA, Bonferroni *post hoc *analyses.

Cell genotoxicity was always found to be not different from negative control (Figure 1), with only an exception for fraction A (ø > 2.5 *μ*m) of tunnel 1. In this case, an increase in MN frequency up to 16.0 ± 0.7, with a fold induction of about 1.5 times with respect to negative control, was observed.

For tunnel 2, the great amount in A fraction (ø > 2.5 *μ*m) did not match with any increase in MN frequency.

Tunnel 3 showed a slight increase in MN frequency (16.5 ± 0.7) for fraction A, not statistically significant with respect to its own control, in contrast with the small amount of PM (total 0.986 mg/m^3^). In tunnel 7, for which it was recorded the highest value of the fraction AF (2.038 mg/m^3^), a slight increase in MN frequency was found for fractions B and C, which, however, have showed to be of modest amount.

For all other tunnels, any significant increase in MN frequency was observed, with any correspondence with amount of particulate collected with the Sioutas sampler.

EMS was used as a positive control to verify the sensitivity of MN test. MN frequency in positive control was 23.0 ± 1.4.

NDI values which are a measure of cytostasis [33] were found always not statistically different from negative controls (Table 2). NDI value of EMS showed a slight decrease with respect to the control, and occasionally decrease observed for some fractions appear to be casual.

### 3.4. TEM

**Figure 2:**
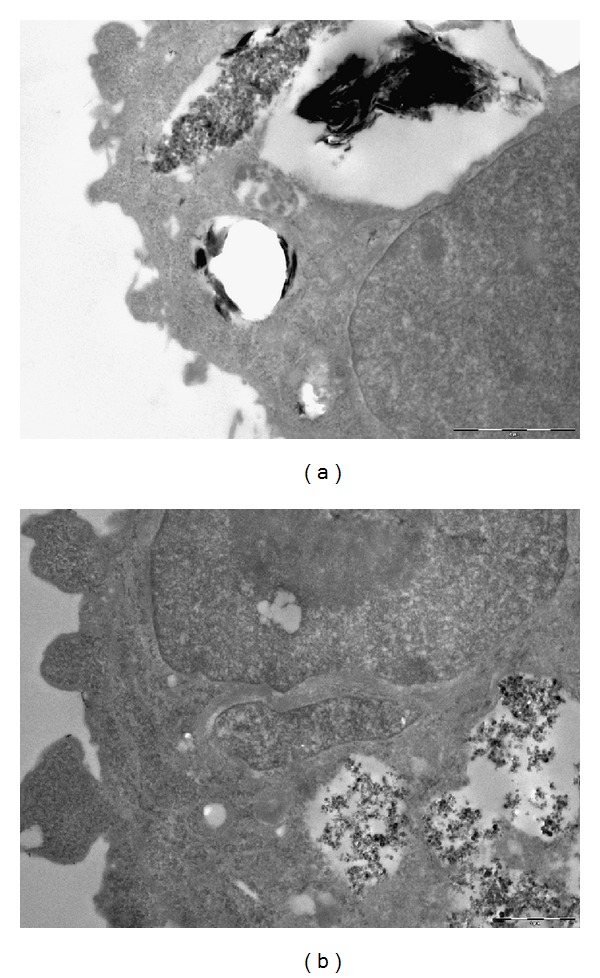
Field emission transmission electron micrograph. Internalization of particulate matter in A549 cells: (a) PM ø > 2.5 *μ*m, 5,000x; bar 10 *μ*m; (b) PM ø < 0.25 *μ*m, 7,500x; bar 5 *μ*m.

TEM images have confirmed the internalization of PM in the alveolar cells (Figure 2).

Interestingly, also fragment of diameter > 2.5 *μ*m could be internalized by A549 cells. In Figure 2(a), a single fragment of vacuolated silica matter is appreciable.  Figure 2(b) shows agglomerates of ultrafine particle matters (PM < 0.25) in vacuolar formation in cytoplasm of A549 cell. Neither agglomerates nor single particles were found in nucleus.

### 3.5. SEM

**Figure 3:**
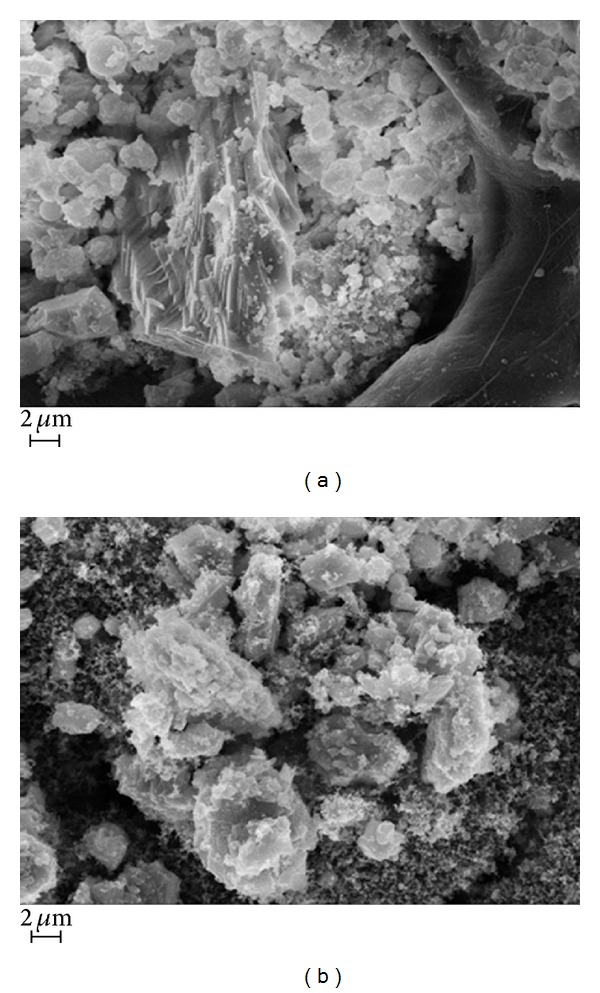
Field emission scanning electron micrograph. Morphological characterization of particulate matter: (a) PM ø > 2.5 *μ*m, 10,000x; (b) PM ø < 0.25 *μ*m, 10,000x; bar 2 *μ*m.

SEM images of particulate of A and AF fractions are showed in Figures 3(a) and 3(b), respectively. Both images are at 10,000x of magnification. In Figure 3(a), it could be appreciated how different particles of different sizes could be found in this fraction. Moreover, the large fragment of siliceous origin is clearly evident at the center of microphotograph. Smaller particles remain adherent to larger ones and are findable in this fraction. Image in Figure 3(b) showed how different in size are particles of fraction AF, with respect to fraction A. Particles of diameter larger than expected could be found in this fraction, this is probably due to turbulent flows, turbulent diffusion, eddies, collisions, and other physical factors that alter the perfect sampling fraction.

## 4. Discussion

All air particles larger than 2.5 *μ*m are classified as coarse particles, whereas particles with an aerodynamic diameter of 2.5 *μ*m or less are characterized as fine particles. Exposure to fine particulate matter (PM) has been found to have adverse effects on cardiopulmonary health, and many population studies have linked exposure to fine PM with increases in hospital admissions and a range of cardiovascular and respiratory diseases and mortality [37]. Although particles with diameters > 1 *μ*m usually remain on the epithelial surface upon their deposition [38] and are subjected to clearance by cough, mucociliary transport, and/or phagocytosis by macrophages, ultrafine particles (UFP: ø < 100 *μ*m, also called “nanoparticles”) seem to penetrate the boundary membranes of the lungs rapidly, a unique feature for insoluble particles [39]. The foreign matter can reach the cytoplasm or the nucleus, and in the worst case, they can cause DNA damage. *In vitro* experiments revealed penetration of UFP into mitochondria of macrophages and epithelial cells that was associated with oxidative stress and mitochondrial damage [40].

PM with diameter lower than 2.5 *μ*m can reach the deep lung and interact with the alveolar epithelium. However, little is known about the alveolar epithelium's ability to internalize inhaled particles. Several studies have reported phagocytosis of PM by alveolar epithelial cells [41].

In our study, silica material was found to be dominant in all size fractions, as identified from the EDX-SEM imaging (microsized amorphous spherical to subspherical particles found in aggregates; Figure 3). When airborne PM reaches environmental concentrations above 1 mg/m^3^, as tested in our study, the particles are mainly inhaled as aggregated and not as single particles [39].

Particles of polygonal morphology, as recognized using EDX-SEM imaging (Figure 3), are identified as silica. Spherical particles are attributed to biogenic processes but may also be in biological forms such as bacteria. Analyses for metals performed with EDX (data not shown) did not reveal presence of heavy metals in the particle samples, thus consisting mainly of siliceous material and, to a lesser extent, of polycyclic aromatic hydrocarbon (PAH). PAH were identified in PM of all the collected fractions and resulted to adhere to silica carrier. Great amount of PM in AF fractions for all tunnels could be explained with massive diesel machines exhaust particles emissions.

The observed differences in terms of genotoxic outcomes were not expected. However, the data obtained in this study (MN) indicate that the genotoxic risk of occupational exposure associated with the tunnel construction work cannot be excluded.

Increase in MN frequencies observed *in vitro* in the micronucleus test was independent of the aerodynamic diameter of the particles. The fraction AF (ø < 0.25 *μ*m) determined a minimal increase in MN frequency in only three samples. Therefore, besides their dimensional aspects, for PM genotoxicity, the chemical composition of airborne particles is probably important.

The highest extent of MN frequency was observed in tunnel 1, where the fraction A, containing particles with diameter above 2.5 *μ*m, resulted significantly higher with respect to negative control. In tunnel 2, where it was registered the highest concentration of total particulate, especially due to fractions A and B, any increase of MN was found.

For tunnels 6 and 7, PM samples showed little effects with the intermediate fractions (B, C and D). Inconclusive results were found for sample from tunnels 4 and 5 where neither increase in MN frequencies nor high concentration of particles was found.

The nuclear division index (NDI) indicated that cell proliferation was not affected in these experiments. Together with negative results obtained in viability testing, NDI data confirmed the absence of cytotoxicity on A549 cells for all the fractions of PM collected. 

Finally, similar numbers and types of diesel-powered machines were used in each tunnel site, and the tunnels were excavated using the same technology. So, characteristics of workplace seemed not to affect the genotoxicity.

Our results are in agreement with those researches that did not reveal a clear effect due to air particles, especially silica [42, 43] that, together with DPM, constitutes the major part of airborne matter in the working environment of tunnels under construction. 

Previous epidemiological studies showed a limited and site-specific genotoxic activity of workplace environment, and the extent of the risk to workers has not yet been assessed [44].

In order to evaluate possibility of internalization of PM in A549 cells, TEM investigations have been performed. Direct [38] or mediated internalization in cytoplasm was documented [41]. Phagosome-mediated internalization seems to be an important mechanism of internalization of particle matter in cells [36].

Our results are in agreement with other studies on A549 cells that proved endocytosis of ultrafine particles in cells [41]. Membrane-bound vacuoles containing large aggregates of particles were observed in all images obtained by transmission microscopy and demonstrated that the majority of particles were localized in vacuoles and not in the nucleus. This could indicate that observed MN increase was most likely a result of indirect mechanisms [39].

In fact, the minimum size for free passage into the nucleus by passive diffusion is 9 nm [45, 46], if signals that favor the formation of the nuclear pore complex are not activated [47].

Some studies have, however, reported nanoparticles in the nucleus, for example, silica nanoparticles (40–70 nm) [48] and silver nanoparticles (6–20 nm) [49]. 

This is in agreement with results of TEM, showing aggregates in the cytoplasm. It is possible to assume that particles when they form agglomerates are not able to pass through the nuclear membrane neither in nuclear pore complexes nor by diffusion and reach DNA and exercise a direct genotoxic effect. 

Sporadic increases in the frequency of micronuclei found in our results can be explained by the contact of the particles with the DNA that could occur during mitosis when the nuclear membrane breaks down, leading to the possibility of direct interactions with DNA. During mitosis, mechanical interference with the microtubules could give rise to aneuploid cells [42]. Yet another possible mechanism is that particles, or intracellular metal ions from particles, can enhance the permeability of the lysosomal membrane, which could lead DNases being released into the cytoplasm and possibly passing into the nucleus, where they could cut DNA [50].

Moreover, translocation depends on other factors different from dimension, but especially the type of material and also nanoparticles as small as 5 nm of diameter was found not internalized in nucleus but remain in cytoplasm [51].

In conclusion, the results of our study show that fractions examined *in vitro* at the concentrations tested did not exert cytotoxic or clear genotoxic effect on pulmonary cells A549, although the particles have been internalized as clearly shown by TEM. This can have two explanations: first, particles at high concentration values, in the order of mg, form aggregates that could have different characteristics and behavior respect to isolated particles; second, the ability of Sioutas multistage cascade sampler that did not discriminate particles with diameters less than 0.25 *μ*m (i.e., nanoparticles), to penetrate the nuclear pore diameter.

## Figures and Tables

**Table 1:** Air particle concentrations in 7 tunnels under construction in an area of Central Italy. For every tunnel, 5 fractions of different diameters were collected.

Tunnel	PM (mg/m^3^)					
	A: ø > 2.5 *μ*m	B: 1.0 < ø < 2.5 *μ*m	C: 0.5 < ø < 1.0 *μ*m	D: 0.25 < ø < 0.50 *μ*m	AF: ø < 0.25 *μ*m	Total
1	0.702	0.15	0.086	0.066	0.322	1.326
2	3.307	1.336	0.713	0.386	0.422	6.164
3	0.549	0.15	0.058	0.054	0.175	0.986
4	0.834	0.287	0.081	0.06	0.347	1.609
5	0.742	0.243	0.105	0.059	0.323	1.472
6	0.369	0.085	0.021	0.021	0.306	0.802
7	0.689	0.202	0.037	0.038	2.038	3.004

**Table 2:** Nuclear division index ± SD in A549 pulmonary cells treated for 24 hours with airborne extracts of tunnel under construction in an area of Central Italy (positive control EMS 2.4 mM value: NDI = 1.67 ± 0.07).

Tunnel	PM (NDI ± SD)					
	A: ø > 2.5 *μ*m	B: 1.0 < ø < 2.5 *μ*m	C: 0.5 < ø < 1.0 *μ*m	D: 0.25 < ø < 0.50 *μ*m	AF: ø < 0.25 *μ*m	Untreated
1	1.82 ± 0.04	1.79 ± 0.04	1.86 ± 0.05	1.87 ± 0.03	1.84 ± 0.04	1.80 ± 0.07
2	1.70 ± 0.06	1.68 ± 0.06	1.70 ± 0.03	1.72 ± 0.07	1.82 ± 0.05	1.73 ± 0.14
3	1.78 ± 0.08	1.85 ± 0.08	1.80 ± 0.05	1.87 ± 0.05	1.81 ± 0.08	1.73 ± 0.14
4	1.73 ± 0.10	1.69 ± 0.12	1.65 ± 0.16	1.69 ± 0.07	1.78 ± 0.05	1.75 ± 0.10
5	1.73 ± 0.07	1.72 ± 0.07	1.73 ± 0.13	1.65 ± 0.06	1.72 ± 0.07	1.75 ± 0.10
6	1.66 ± 0.08	1.72 ± 0.10	1.70 ± 0.06	1.80 ± 0.05	1.71 ± 0.014	1.71 ± 0.12
7	1.74 ± 0.17	1.73 ± 0.08	1.75 ± 0.14	1.71 ± 0.11	1.62 ± 0.07	1.71 ± 0.12
